# Spatial and Temporal Habitat Segregation of Mosquitoes in Urban Florida

**DOI:** 10.1371/journal.pone.0091655

**Published:** 2014-03-12

**Authors:** Paul T. Leisnham, Shannon L. LaDeau, Steven A. Juliano

**Affiliations:** 1 Department of Environmental Science and Technology, University of Maryland, College Park, Maryland, United States of America; 2 Cary Institute of Ecosystem Studies, Millbrook, New York, United States of America; 3 School of Biological Sciences, Illinois State University, Normal, Illinois, United States of America; Virginia Tech, United States of America

## Abstract

Understanding mechanisms fostering coexistence between invasive and resident species is important in predicting ecological, economic, or health impacts of invasive species. The non-native mosquitoes *Aedes aegypti* and *Culex quinquefasciatus* have been resident in the southeastern United States for over a century. They coexist at some urban sites with the more recent invasive *Aedes albopictus,* which is usually superior in interspecific competition. We tested predictions of temporal and spatial habitat segregation that foster coexistence of these resident species with the superior invasive competitor. We measured spatial and temporal patterns of site occupancy and abundance for all three species among standard oviposition traps in metropolitan Tampa, Florida. Consistent with the condition-specific competition hypothesis, *A. albopictus* and *A. aegypti* abundances were greater and *C. quinquefasciatus* abundance was lower late (September) versus early (June) in the rainy season, and the proportional increase of *A. albopictus* abundance was greater than that of *A. aegypti*. These results are postulated to result from greater dry-season egg mortality and associated greater rainy-season competitive superiority of larvae of *A. albopictus,* followed by *A. aegypti,* and *C. quinquefasciatus.* Spatial partitioning among landscape variables was also evident among species, with *A. albopictus* more likely to oviposit across a range of open grass landscapes whereas *A. aegypti* were mostly restricted to cemeteries. *Culex quinquefasciatus* showed a shift in abundance from cemeteries early in the rainy season to developed areas characterized by built environments with large proportions of impervious surfaces late in the rainy season, where *A. albopictus* was not in its highest abundance. These results suggest that both temporal and spatial variation, and their interaction, may contribute to local coexistence between *Aedes* and *Culex* mosquito species in urban areas.

## Introduction

Ecological theory and empirical work indicate that competition often results in competitive exclusion when resources are limited. However, competitive exclusion may be avoided via a number of mechanisms, including differential resource use (e.g., [1]), temporally varying condition-specific competition and the storage effect (e.g., [2]), and spatial resource partitioning (e.g., [2]). Understanding mechanisms of coexistence is particularly interesting in the context of biological invasions. Competitively superior invasive species may impact the distribution and abundance of resident species without causing their extinction over the entire introduced range (e.g., [1]). Identifying mechanisms contributing to local or spatially patterned coexistence of invasive and resident species is important for predicting future ecological, economic, or health impacts of species invasions.

The Asian Tiger mosquito, *Aedes albopictus* (Skuse), is native to Asia and has invaded North and South America, Europe, and Africa in the past three decades (see [3] and references therein). This species is well studied [4], and provides us with an opportunity to determine whether patterns of coexistence with natives in its new range are consistent with any of these coexistence mechanisms. *Aedes albopictus*, and its competitors, such as *Aedes aegypti* (L) and *Culex quinquefasciatus* Say, a member of the *Culex pipiens* complex, utilize water-holding natural (e.g., tree holes, plant axils) and artificial (e.g., tire casings, trash, bird baths) container habitats for their egg and larval stages. *Aedes aegypti* and *C. quinquefasciatus* invaded the Americas from Africa during the 15–17th centuries and the 19th century, respectively [5]. Both species have widely colonized container habitats in the southeastern United States, and are now widely considered non-native resident species with regards to the more recent *A. albopictus* invasion [4]. Biting females that emerge from these aquatic container habitats are principal vectors of arboviruses worldwide, including dengue, chikungunya, yellow fever, La Crosse encephalitis, and West Nile virus [6]–[9], and understanding mechanisms of coexistence among these vectors is of human health importance.

Most laboratory and field studies (see [10], [11] and references therein) convincingly show that larval *A. albopictus* are superior competitors for resources over *A. aegypti* and members of the *C. pipiens* complex, and *A. aegypti* appears to have a clear competitive advantage over *C. pipiens pipiens* [12]–[14]. However despite this competitive hierarchy, inferior competitors *A. aegypti* and *C. quinquefasciatus* often coexist with invading *A. albopictus* in the southeastern USA, and are sometimes the most common container mosquitoes [15], [16]. A number of hypotheses could explain the persistence of *A. aegypti* and *C. quinquefasciatus* after the invasion of *A. albopictus*. Here we use a spatially and temporally explicit field dataset to evaluate two prominent hypotheses: condition-specific competition and spatial partitioning.

Differential mortality among development stages affects R*, the equilibrium resource abundance necessary to produce zero net population growth [1], which determines competitive advantage [1]. Abiotic conditions that differentially affect mortality, even in non-competing life-stages, such as eggs, can alter the outcome of interspecific competition by differentially changing species’ R*s [17], [18]. Condition-specific competition occurs when the outcome of competition is altered or reversed under different abiotic conditions [19]. When there are temporal or spatial fluctuations in abiotic environments, and species with environmentally resistant life-cycle stages respond differently to those environments, the competitive outcome can be altered and coexistence can result [1], [2].

Condition-specific competition between *A. albopictus* and *A. aegypti* has been observed in the laboratory [17]. Experimental manipulation of container drying regime in laboratory colonies shows that in dry conditions, *A. albopictus* suffers a greater interspecific density effect than *A. aegypti,* whereas under wetter conditions, *A. aegypti* suffers a greater interspecific density effect than *A. albopictus* [17]. Under dry conditions, *A. albopictus* eggs suffer greater mortality than do *A. aegypti* eggs [17]. Drought is a strong environmental influence where these species coexist in the southern part of Florida, which experiences a distinct cycle of rainy (June–September) and dry (October–May) seasons (National Oceanic and Atmospheric Administration: URL: http://www.ncdc.noaa.gov/cdo-web/search). Abundances of both species in field containers in south Florida support condition-specific competition, with abundances being higher late in the rainy season (i.e., September) vs. early in the rainy season (i.e., June) [20]. The proportional increase of *A. albopictus* from the early to late rainy season is greater than that of *A. aegypti* presumably due to higher dry-season egg mortality and strong rainy-season competitive superiority of larval *A. albopictus* [20]. A single field study investigating competition between these species during rainy and dry seasons did find seasonal differences in the intensity of competition, but competitive effects favoring *A. albopictus* were evident only in the rainy season [21]. Both species responded in similar ways to the dry season, and evidence for a role for desiccation induced mortality was absent [21]. More intense competition during the rainy season was likely due to differences in detritus resource availability, which was greater during the dry season. Thus, although condition-dependent coexistence of *A. albopictus* and *A. aegypti* remains plausible, there is a clear need for additional testing of this hypothesis.

Competition between *C. quinquefasciatus* and *Aedes* mosquitoes in varying wet-dry regimes has not been tested in the laboratory but we expect that competitive success of *C. quinquefasciatus* would be highly dependent on wet conditions because it oviposits by depositing floating rafts of eggs in existing aquatic habitats. Thus, unlike *A. albopictus* and *A. aegypti*, which oviposit desiccation-tolerant eggs on the insides of containers prior to flooding, ovipositing *C. quinquefasciatus* require existing aquatic habitats, and thus are more strongly limited by their availability. Additionally, habitat persistence to support *Culex* egg hatching and larval eclosion directly affects *Culex* mortality and adult production. Dry conditions are expected to cause greater mortality of *C. quinquefasciatus* eggs and larvae compared to *Aedes* eggs and larvae because *Aedes* larvae are only at risk from habitat drying if eggs hatch after a flooding event. For coexistence between *A. albopictus* and *A. aegypti* to occur under these circumstances, environments must fluctuate between conditions favoring the different species and there must be resistant life stages (e.g., dormant eggs) that persist through times when a species is at a disadvantage [2]. For *Culex* to coexist with both the *Aedes* species there must be sufficient rainfall and favorable climate conditions to allow habitats to persist long enough for both oviposition and immature development.

Poorer competitors may also escape exclusion if there is resource partitioning in space [22], [23]. Spatial partitioning is most likely to arise due to greater availability of habitats and attraction of competitors to different environmental conditions [2], [24]. Prior studies have shown differential habitat utilization of competing mosquitoes among land use types [25]–[27]. For example, a study of the oviposition ecology of *A. albopictus* and *A. aegypti* in Rio de Janeiro, Brazil and Boca Raton, Louisiana, USA showed that in general *A. aegypti* was most prevalent in highly urbanized areas and *A. albopictus* in rural, suburban and vegetated urban areas, but that abundances of both species were similar in suburban areas [26]. Spatial partitioning among land use types likely results from direct effects of landcover on both aquatic and terrestrial habitat quality and population success, and from behavioral habitat choice, but few studies have rigorously examined the specific relationships and mechanisms explaining the distribution, abundance and co-occurrence of mosquito species in a heterogeneous urban landscape [25], [27].

In this paper, we evaluate the importance of seasonal condition-specific competition and habitat segregation hypotheses in explaining temporal and spatial distributions and co-occurrence of immature *A. albopictus*, *A. aegypti,* and *C. quinquefasciatus* across three cities (Tampa, Bradenton, and Palmetto) in greater metropolitan Tampa, Florida, USA. Metropolitan Tampa is ideal for such tests because it has a diverse range of habitats within a few kilometers of one another, and because these species have coexisted at some sites in metropolitan Tampa for over 15 years (G.F. O’Meara, unpublished data), suggesting stability. A prior study of mosquito oviposition ecology in Tampa found evidence for habitat segregation and condition specific competition between *A. aegypti* and *A. albopictus* among broad classes of residential, industrial, and commercial land during the rainy season [20]. However, [20] had three important limitations that we address in this paper. First, [20] did not consider *C. quinquefasciatus* despite this species being the third most common mosquito collected from oviposition traps, and potentially having a different pattern of abundance between dry vs. wet conditions than *Aedes*. Second, [20] did not evaluate how habitat partitioning among species may vary between the early vs. late rainy season. Third, although past work has indicated that cemeteries containing flower vases may act as important habitat for mosquitoes in Florida (e.g., [15], [20], [28]), [20] only compared cemeteries with randomly chosen non-cemetery urban areas rather than the full range of land cover types that exist within diverse urban landscapes.

Our investigation in this paper directly builds on [20] by re-sampling the same study areas, evaluating habitat partitioning among *Aedes* and *Culex* mosquitoes across different land uses, and explicitly comparing cemeteries with other land uses. We evaluate occupancy, abundance, and co-occurrence across different land cover types at the beginning of the rainy season (June) and after several months of wet conditions (September). As with [20] and other studies that sample mosquitoes among landscapes (e.g., [25], [26], [27]), our investigation is observational and can only provide a snapshot of habitat utilization in the field. However, our hypotheses explaining coexistence among species generate testable predictions about the distribution and abundance of these mosquitoes in a heterogeneous urban landscape.

1. Condition-specific competition predicts that all species occupy a greater proportion of containers and are more abundant per container late in the rainy season but that there are greater proportional increases in *A. albopictus* and *C. quinquefasciatus* from early to late rainy season, compared to the dry season, due to high dry-season (October–May) egg mortality and loss of oviposition habitat, respectively, and strong rainy-season (June–September) competitive superiority of *A. albopictus* and greater available habitat for *C. quinquefasciatus*.
2. The spatial habitat segregation hypothesis predicts co-occurrence if species occupy and are more abundant in different habitats defined by landuse. Competitive superiority of *A. albopictus* predicts persistence of *A. aegypti* and *C. quinquefasciatus* in urban areas if they are able to exploit different habitats compared to *A. albopictus,* especially in the rainy season when *A. albopictus* is expected to be most common.

These hypotheses are not mutually exclusive and may both contribute to coexistence among *A. albopictus, A. aegypti,* and *C. quinquefasciatus.*

## Materials and Methods

Three areas within metropolitan Tampa (Tampa, Hillsborough County [lat. 27.9°, long. −82.4°]; St. Petersburg, Pinellas County [lat. 27.8°, long. −82.6°; and Bradenton, Manatee County [lat. 27.5°, long. −82.4°), all separated by Tampa Bay, were selected for study. Southern Florida experiences greater rainfall and temperatures in the summer rainy season (June–September) compared to the dry season (October–May) (National Oceanic and Atmospheric Administration: URL: http://www.ncdc.noaa.gov/cdo-web/search). Mean monthly rainfall and daily average temperature for the three meteorological stations closest to our study sites reflect this pattern for both the period from 1981–2010 (Dry season: 66.4 mm, 20.5°C; Rainy season: 200.1 mm, 28.1°C), the dry season before our sampling in 2008 (55.8 mm, 21.2°C), and the rainy season in 2008 (162.0 mm, 28.1°C).

Each area consisted of 60 km^2^ divided into 60 1-km^2^ grid cells. These areas were selected because they have residential, commercial, and industrial land uses [29], a high density of human-created structures, diverse vegetation types, and a number of cemeteries with suitable mosquito habitats (Tampa, *n = *10; St. Petersburg, *n* = 5; and Bradenton, *n* = 4) that were used by [20]. Within each area, we randomly selected the centers of a subset of urban cells (Tampa, *n = *34; St. Petersburg, *n* = 34; and Bradenton, *n* = 32) and each cemetery as sampling sites to give a total of 100 urban and 19 cemetery sites. Other sites with high densities of container habitats, such as tire yards, are present in each area but preliminary observations suggested that few had long standing piles of tires outside that would fill with water and provide larval mosquito habitat. Thus, we decided not to survey tire yards in this study.

Consistent with [20], we used oviposition traps (ovitraps) to sample mosquito populations. Ovitraps allow the rigorous testing of relationships between mosquito oviposition ecology and broad landscape-scale variables by standardizing individual sampling container. Ovitraps are widely used to sample *Aedes* oviposition in time and space (e.g., [25]–[27], [30], [31]), and are effective at sampling *Culex* when baited with resource (hay, grass, leaves etc) infusion (e.g., [20], [32], [33]). The occurrence of eggs in ovitraps is considered a sensitive indicator for identifying the presence of many mosquito species [34], [35], and more sensitive even when compared to adult collection methods [36], [37]. The occurrence and abundances of *Aedes* from ovitraps has been positively associated with rainfall and temperature patterns [38], [39], and because of their strong sensitivity, ovitraps are considered particularly effective at sampling mosquitoes during unfavorable seasons (i.e., dry seasons and/or lower temperatures) [36]. Abundances from ovitraps can be less reliable at predicting oviposition intensity and adult densities than other methods [35], but they are more affordable, and thus more highly replicable than adult traps, and easier to sample than existing resident containers [30], making them ideal to compare broad trends in mosquito presence and abundance across large numbers of sites and between seasons, as in this study.

In the early rainy season (June) in 2008, 3 ovitraps were placed in the shade at each site. Ovitraps were placed at ground level and within 20 m of one another (357 total traps). Ovitraps consisted of black plastic cups (400 ml), with holes drilled 4 cm from the base to prevent flooding and hatching of *Aedes* eggs. Ovitraps were lined with seed germination paper (Nasco Science®), filled with 225 ml deionized water, and baited with an additional 25 ml of grass/oak leaves infusion (72 g senescent live oak (*Quercus virginiana*) leaves and 36 g Zoisa grass in 5.4 L of deionized water for 3 days). Ovitraps in alternating counties were set out over six days, with 14–20 stations being provisioned with traps on any particular day. After 7 days, ovitraps were collected and all larvae identified. *Culex* egg rafts from each ovitrap were collected and stored on water to allow eggs to hatch. Germination paper from each ovitrap was also stored in humid conditions for 10 days then immersed in nutrient broth solution to hatch eggs. Numbers of field-collected larvae and pupae and laboratory-hatched larvae were summed by species. Larvae were reared to 4th instar to facilitate identification and larvae and pupae stored in 70% ethanol for later examination in the laboratory. During the late rainy season (September) in 2008, we repeated our sampling using this same protocol. When collecting ovitraps in June we measured the remaining water in each trap and recorded if the trap had dried completely. Preliminary analyses of June data indicated no relationships between water volume and mosquito abundances among ‘wet’ traps; thus we only scored traps as ‘dry’ or ‘wet’ in September collections. Field collected larvae included no endangered or protected species, and no prior permissions were required to access study sites or collect mosquitoes.

The relatively small size and inconspicuous color of these ovitraps was expected to minimize chances of them being disturbed in areas of relatively high human activity, and has been used in previous oviposition surveys in urban landscapes [20], [40]. Twelve and 10 traps were disturbed across all three areas in June and September respectively, including all three traps at one site in St. Petersburg in September, thus these cups were removed from all analyses.

### NLCD and Land Use Data

A subset of our sites from each city was purposely located in cemeteries. Additionally, we examined land cover around each site at two spatial scales (50-m and 200-m buffers). These distances were chosen *a priori* to represent the local development site characteristics (50 m) and characteristics of the wider landscape within the dispersal distances of all species (200 m). Data from the National Land Cover Database (NLCD, 2001) were downloaded at the USGS Landcover Institute (available online at: http://landcover.usgs.gov/). We tabulated the number of pixels in each NLCD-defined land cover class and used the standardized proportional area in each class as explanatory variables in analyses described below. Given that all of our sites were located in a metropolitan area, we selected three NLCD available land cover class categories that best described the variation seen among our sites for the analyses: High Intensity Developed (impervious surfaces >79%), Open Space (predominantly lawns and parkland), and Wetland cover (including both woody and herbaceous). A fourth category, Low Intensity Developed was also common but was significantly correlated with each of the other three and thus, was not used in this analysis.

### Statistical Analyses

We evaluated the relative abundance of mosquito larvae across 119 sites in three cities from two sampling dates. We were specifically interested in examining how relative abundance and multiple species occurrence (co-occurrence at a site) varied with seasonality and with land cover. We used the species-specific mean number (rounded to nearest integer) of larvae from the replicate traps at each site as our dependent variable in the analyses described below.

Count data are generally assumed to follow a Poisson distribution, although large numbers of zeros can invalidate the Poisson assumption of equal mean and variance. We formally compared a standard Poisson linear regression with a zero-inflated Poisson (ZIP) model. In the ZIP model, zeros can be generated both due to the Poisson sampling structure (e.g., random zeros) and due to site unsuitability. We used a coupled GLM approach (e.g., [41], [42]) to estimate both the proportion of zeros that could best be attributed to site unsuitability (i.e., latent site suitability parameter) vs. the (Poisson) structural zeros and a generalized linear regression with explanatory variables describing both processes. In this model, the probability of a Poisson zero is δ_is_ for species *s* at a site *i* and occurs with probability ρ_is_:
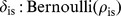
and logit(ρ_is_) is a species-specific function of sampling season.

When the latent ‘site suitability’ estimate is 1, (i.e., zeros are generated from a Poisson sampling distribution), then observed counts (*Y_is_*), including zeroes, are conditionally independent with unknown parameters λ_is_ representing the true abundance of species *s* at site *i.* We assumed that each of these conditional distributions is Poisson with mean 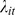:
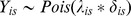



Then, for all *i* = 1,…,N sites and *s = *1,…,S species,
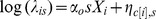



Relative abundance is thus, a function of site suitability, the explanatory variables in matrix ***X*** (as defined below), and a hierarchical structure, 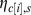, which allows for correlation among species-specific abundances within a city beyond what is explained by components of ***X***.

We used the model framework described above to test each of our hypotheses, testing specifically the relative importance of seasonal and land use effects as predictors of population abundances and co-occurrences of the two *Aedes* species and *Culex quinquefasciatus.* We ran an initial model where the matrix X included the vector of ones, and three binary indicator variables for month and presence of each of the other two species. We ran the model again where X included a vector of ones, a binary indicator variable for month and cemetery location, and the three land cover variables described in the previous section at both 200 and 50 meter scales. Although we could have integrated the seasonal and co-occurrence variables into the land cover analysis, we chose to focus on two simpler models with rapid convergence that more directly addressed our separate hypotheses about seasonality (condition-dependent) and habitat segregation. Results in the text are shown as the mean followed by the 95% credible interval derived from the posterior distribution for each parameter. We evaluated the fit of the ZIP model relative to a simpler Poisson regression that does not include zero inflation but uses the same definition of λ_is_.

Bayesian inference requires prior distributions be assigned to all unknown parameters and we used standard conjugate distributions in each case [43]. 1,…, *P* parameters (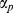) describing covariate effects in the regression equation was given a vague (flat) Gaussian prior distribution with mean 0 and large variance (1000). For each city *c*, 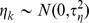 and 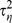 was sampled from a relatively uninformative inverse gamma distribution with parameters (0.01, 0.01). Model parameters were estimated by simulating from the joint posterior distribution of all unknown parameters using a Markov chain Monte Carlo (MCMC) algorithm implemented using the WinBUGS software [43]. We evaluated model fit in three ways. DIC is a measure of how expanding or decreasing model structure changes the prediction accuracy of the model, with lower DIC values (>5 units difference) representing the preferred model [44]. We further evaluated model fit with and without zero-inflation by comparing the proportion of zeros and overdispersion using model predictions and raw data. We used the overdispersion index: OD.i = Var(y)/E(y), where y = either observed or model predicted count data. For each of the three focal species, the zero-inflated Poisson models provided a better fit to data than a simple Poisson structure (Table 1).

**Table 1:** Comparisons between simple Poisson structure and zero-inflated Poisson (ZIP) models for describing *Aedes albopictus, Aedes aegypti, and Culex quinquefasciatus* abundances.

	Proportion Zeros			Overdispersion Index			DIC*
	Observed	Poisson	ZIP	Observed	Poisson	ZIP	Poisson
*A. albopictus*	0.76	0.379	0.79	22.25	5.42	13.89	+973
*A. aegypti*	0.51	0.046	0.53	23.59	2.04	9.61	+1424
*C. quinquefasciatus*	0.59	0.001	0.58	101.60	22.21	53.57	+8233

*DIC, deviance information criterion, shown as deviation from smallest DIC for each species.

## Results

*Aedes aegypti*, *Culex quinquefasciatus*, and *A. albopictus* were the most widespread species sampled, being collected from 25.0% (173/692), 20.5% (142/692), and 12.1% (84/692) of ovitraps respectively. When pooling abundances across all ovitraps, *C. quinquefasciatus* was the most common species collected (17,844/22,360 individuals), followed by *A. aegypti* (3,086/22,360) and *A. albopictus* (1,430/22,360). *Culex nigripalpus, Aedes triseriatus* and *Toxorhynchites rutilus* were also collected from ovitraps but constituted less than 1% of the total number of individuals. A total of 59 ovitraps, occurring at 32.8% of sample sites (39/119) were dry in the early rainy season (June), including all ovitraps at 6 sites (5.0%). Dry ovitraps in the early rainy season were most common at sites surrounded by more high intensity developed cover (r = 0.217, t = 2.40, p = 0.017), and not related to any other land cover variables (p>0.050). The 6 sites with all dry ovitraps were removed from further analyses.

The proportion of sites *A. aegypti* and *C. quinquefasciatus* occupied decreased between the early and late rainy season, whereas the proportion of sites with *A. albopictus* increased over this same period. *Aedes albopictus* was nearly twice as likely to co-occur at sites with *A. aegypti* in the late rainy season (10.1%, 12/119) relative to the early rainy season (5.3%, 6/113), whereas its co-occurrence with *C. quinquefasciatus* was less common and did not vary (3.5%, 4/113 vs. 3.4%, 4/119) (Table 2). *Aedes aegypti* and *C. quinquefasciatus* co-occurred at 31.0% (35/113) sites in the early rainy season but only 1.7% (2/119) sites in the late rainy season (Table 2).

**Table 2:** Numbers and proportions of sites and individual oviposition traps (in parentheses) occupied by immature of *Aedes albopictus* (ALB), *A. aegypti* (AEG), and *C. quinquefasciatus* (CQ) in cemeteries and urban sites in Palmetto, St. Petersburg, and Tampa, FL, in the early (June) and late (Sept.) rainy seasons.

	Number of sites	ALB only	AEG only	CQ only	ALB+AEG	ALB+CQ	AEG+CQ	ALB+AEG+CQ	None	Proportion ALB	Proportion AEG	Proportion CQ
Early rainy season (June)												
Cemeteries	16 (37)	0 (1)	4 (7)	1 (8)	0 (0)	3 (4)	3 (5)	2 (1)	3 (11)	0.13 (0.16)	0.56 (0.35)	0.56 (0.49)
Palmetto	32 (88)	0 (0)	3 (9)	3 (14)	3 (5)	0 (1)	14 (17)	3 (1)	6 (41)	0.19 (0.08)	0.72 (0.36)	0.63 (0.38)
St. Petersburg	32 (76)	0 (1)	6 (12)	6 (16)	0 (0)	1 (1)	12 (12)	1 (0)	6 (34)	0.06 (0.03)	0.59 (0.32)	0.63 (0.38)
Tampa	33 (85)	0 (6)	5 (14)	7 (13)	3 (4)	0 (0)	6 (12)	6 (4)	6 (32)	0.27 (0.16)	0.61 (0.40)	0.58 (0.34)
Early total	113 (286)	0 (8)	18 (42)	17 (51)	6 (9)	4 (6)	35 (46)	12 (6)	21 (118)	0.19 (0.10)	0.63 (0.36)	0.60 (0.38)
Late rainy season (Sept)												
Cemeteries	19 (57)	4 (7)	4 (7)	1 (2)	2 (5)	2 (2)	0 (0)	2 (1)	4 (27)	0.53 (0.26)	0.53 (0.23)	0.26 (0.09)
Palmetto	33 (93)	2 (14)	8 (14)	5 (6)	3 (3)	0 (0)	0 (1)	3 (2)	12 (62)	0.24 (0.20)	0.42 (0.22)	0.24 (0.10)
St. Petersburg	33 (95)	2 (12)	7 (12)	7 (12)	0 (0)	2 (0)	2 (0)	0 (0)	13 (67)	0.12 (0.13)	0.27 (0.13)	0.33 (0.13)
Tampa	34 (102)	6 (15)	7 (15)	5 (6)	7 (9)	0 (0)	0 (0)	2 (1)	7 (61)	0.47 (0.25)	0.44 (0.25)	0.21 (0.07)
Late total	119 (347)	14 (48)	26 (48)	18 (26)	12 (17)	4 (2)	2 (1)	7(4)	36 (217)	0.39 (0.20)	0.31 (0.20)	0.26 (0.10)

Only totals for cemeteries among cities are shown.

### Occurrence and Abundance: Seasonal Patterns

The relative abundance of *A. albopictus* and, to a lesser degree, *A. aegypti* increased from the early to the late rainy season across occupied sites, while *C. quinquefasciatus* abundances declined during this same time period (Table 3). Consistent with the data summary in Table 2, the model estimates that *A. aegypti* and *C. quinquefasciatus* occupied a larger proportion of sites across both sample periods (0.47 and 0.42, respectively) relative to *A. albopictus* (0.22, Table 3). Our analysis suggests asymmetric influence of co-occurrence on species abundances. The relative abundance of *A. aegypti* was an estimated 73.3% and 41.9% more abundant across sites where *C. quinquefasciatus* or *A. albopictus* were present, respectively (Table 3), although occurrence of *Ae. aegypti* was not predictive of either *Culex* or *A. albopictus* abundance. Similarly, *A. albopictus* was an estimated 63.2% more abundant when *C. quinquefasciatus* larvae were present (Table 3), although *C. quinquefasciatus* abundance was on average 6.8% lower at sites where *A. albopictus* was present.

**Table 3:** Posterior parameter estimates (with 95% CI) for models describing variation in relative abundances of *Aedes albopictus, Aedes aegypti,* and *Culex quinquefasciatus* by season, when each of the other species was present.

	*Aedes albopictus*			*Aedes aegypti*			*Culex quinquefasciatus*		
Parameter	Mean	2.5%	97.5%	Mean	2.5%	97.5%	Mean	2.5%	97.5%
Intercept	**0.99**	**0.44**	**1.43**	**1.48**	**1.25**	**1.76**	**4.27**	**4.09**	**4.49**
Month	**1.42**	**1.15**	**1.71**	**0.82**	**0.68**	**0.96**	−**0.68**	−**0.74**	−**0.61**
*A. albopictus* presence	–	–	–	**0.35**	**0.22**	**0.48**	−**0.07**	−**0.14**	−**0.02**
*A. aegypti* presence	−0.05	−0.25	0.15	–	–	–	0.03	−0.02	0.08
*C. quinquefasciatus* presence	**0.49**	**0.3**	**0.68**	**0.55**	**0.41**	**0.68**	–	–	–
Presence	**0.22**	**0.16**	**0.27**	**0.47**	**0.41**	**0.54**	**0.42**	**0.36**	**0.48**

Mean values less than 0.0 for Month indicate a decline in abundance between early and late rainy season. Bold-face indicates that the posterior distribution (95% CI) does not include zero.

### Relative Importance of Land Cover

The importance of cemetery habitat varied by species and between the early and late rainy season. Cemetery habitat was positively associated with abundance of *A. albopictus* and *A. aegypti* in the late rainy season (September), whereas both species were less abundant at cemetery sites relative to other land cover categories during the early rainy season (June) (Table 4). *Culex quinquefasciatus* showed the opposite response, with cemetery sites being more important predictors of abundance in June and negatively associated with abundance in September (Table 4). There was no improvement in model fit when land cover characteristics at the 50-m scale were added to the base model that included month and cemetery indicator variables. If cemetery was excluded, Open Cover Developed area within a 50-m buffer was a positive predictor of *C. quinquefasciatus* in the early rainy season (0.427, 95% CI (0.339, 0.515) and of *A. albopictus* in the late rainy season (3.188, 95% CI (1.838, 4.708)).

**Table 4:** Posterior parameter estimates (with 95% CI) for models describing the relative importance of land cover (200-m buffer) in explaining variation in larval abundance across the rainy season.

	*Aedes albopictus*		*Aedes aegypti*		*Culex quinquefasciatus*	
Parameter	Early	Late	Early	Late	Early	Late
Intercept	−0.431		1.393		0.821	
	(−3.385, 1.783)		(−0.133, 2.641)		(−0.335, 1.580)	
Cemetery	−**1.665**	**2.161**	−**1.275**	**1.689**	**0.513**	−**1.128**
	**(**−**3.053,** −**0.617)**	**(1.053, 3.551)**	**(**−**1.659,** −**0.908)**	**(1.263,** −**2.143)**	**(0.416, 0.615)**	**(**−**1.464,** −**0.802)**
Wetland	0.312	2.158	−**2.286**	−**3.759**	−0.367	−**0.983**
	(−3.270, 3.436)	(−1.355, 5.856)	**(**−**3.777,** −**0.887)**	**(**−**6.160,** −**1.435)**	(−0.759, 0.073)	**(**−**2179,** −**0.061)**
High Intensity Developed	1.066	−0.039	**0.959**	−**2.051**	−0.084	**0.753**
	(−0.969, 2.938)	(−2.321, 2.329)	**(0.021, 1.915)**	**(3.223,** −**0.810)**	(−0.379, 0.221)	**(0.289, 1.214)**
Open Cover Developed	−0.658	**2.755**	**1.544**	−**2.289**	−**0.320**	**1.325**
	(−2.638, 1.361)	**(0.684, 4.984)**	**(1.008, 2.093)**	**(**−**3.000,** −**1.598)**	**(**−**0.500,** −**0.128)**	**(0.759, 1.914)**

Parameters describing the association with each land cover type were estimated separately for samples from early (June) and late (Sept.) rainy season. Bold-face indicates that the posterior distribution (95% CI) does not include zero.

In addition to cemeteries, High Intensity Developed area and Open Cover Developed area were important predictors of abundance for at least one of the species at the 200-m scale (Table 4). Wetland area was not significantly positively associated with any species’ abundances but was negatively associated with *A. aegypti* and *C. quinquefasciatus* (Table 4). Both High Intensity Developed area and Open Cover Developed area were positively associated with *A. aegypti* early in the rainy season but by September, cemetery locations were the predominant habitat for this species (Table 4). Relative abundance of *A. albopictus* was not positively associated with any habitat variables in the early rainy season but was greatest at cemeteries and at sites with more Open Cover Developed area in the late rainy season (Table 4). By contrast to the two *Aedes* species, *C. quinquefasciatus* shifted from an early-season cemetery focus to greater abundances at sites with greater High Intensity Developed and Open Cover Developed area by September (Table 4).

All models included the latent site-suitability parameter and a seasonal effect was estimated. Zero counts for both *A. aegypti* and *C. quinquefasciatus* were less likely to be a random Poisson zero and more likely to be due to unsuitable habitat in late versus early season (seasonal effect: *A. aegypt*i −0.862, 95% CI (−1.390, −0.343); *C. quinquefasciatus*: −1.389 95% CI (−1.946, −0.839)), while unsuitable habitat was a more likely explanation for zeros in the early season for *A. albopictus* (0.658, 95% CI (0.056, 1.279)).

## Discussion

The results of this study show clear patterns in the seasonal and spatial distributions of *A. albopictus, A. aegypti,* and *C. quinquefasciatus* in metropolitan Tampa that are consistent with seasonal condition-specific competition and habitat segregation as mechanisms contributing to species coexistence. These results also highlight important interactions between these two mechanisms that can only be explored when both space and time are considered explicitly, as we have done in this study. The persistence of *A. aegypti* and *C. quinquefasciatus* in the presence of the competitively superior *A. albopictus* may be explained by seasonal condition-specific competition, if there are seasonally-related differences in survival among the species. We predicted *A. albopictus* would show a greater increase in the proportion of sites occupied and per site abundance during the rainy season than *A. aegypti,* due to its high dry season egg mortality and strong rainy-season competitive superiority. Our results were consistent with this prediction, with both *Aedes* species increasing in per site abundance from early to late season, but with *A. albopictus* abundance increasing almost twice as much on average relative to *A. aegypti* abundance (Table 3). While the proportion of occupied sites increased from early to late rainy season for *A. albopictus,* they actually decreased for *A. aegypti,* suggesting that rainy-season competition from *A. albopictus* had a particularly severe negative effect on the distribution of *A. aegypti.* While both *Aedes* species appeared to favor cemetery sites later in the rainy season (which may explain why *A. aegypti* abundances were generally greater when *A. albopictus* was also present at a given site, Table 3), *A. albopictus* was also abundant in sites characterized by greater Open Developed Cover. This NLCD category includes golf courses, open parks, lawns and would also cover cemeteries. These findings suggest that *A. albopictus* was more likely to oviposit in wet sites across the range of open grass categories, whereas *A. aegypti* was more restricted to habitat specific to cemetery sites.

Laboratory tests have shown that *A. albopictus* is a superior competitor to members of the *C. pipiens* complex [13], [45], and we found *C. quinquefasciatus* abundance to be lower at sites with *A. albopictus* than those without the invader. *Culex quinquefasciatus* is more sensitive to dry conditions than are *Aedes.* Therefore, we expected *C. quinquefasciatus* to experience high dry-season mortality resulting in low abundance early in the rainy season, and that abundances would increase during the rainy season as more water-filled container habitats become available. However, as with *A. aegypti, C. quinquefasciatus* occupied a greater proportion of sites early in the rainy season, and its site occupancy and abundance declined from the early to late season. These results are inconsistent with condition-specific competition as the main mechanism of *C. quinquefasciatus* coexistence with *A. albopictus* and *A. aegypti. Culex quinquefasciatus* did however demonstrate a seasonal shift in habitat preference that may be important for its persistence with these *Aedes* species, and that is consistent with a spatial habitat segregation mechanism of coexistence by season. The associations between *C. quinquefasciatus* and land cover characteristics were seasonally distinct from both *Aedes* species, with a shift in abundance from cemetery sites early in the rainy season to both High Intensity Developed and Open Cover Developed areas by the late rainy season (Table 4). High Density Developed areas include heavily built environments such as some commercial and residential areas where impervious cover is high.

Numerous studies have shown that the seasonal patterns of *A. aegypti* and *A. albopictus* abundances are linked with local rainfall (e.g., [31], [46], [47]), and these patterns have been interpreted as being a result of dry-season egg mortality and rainy-season competitive ability [17], [21]. The southern peninsula of Florida has predictable seasonal differences in rainfall, with high total precipitation from frequent rain showers from May through September (rainy season) and low total precipitation from infrequent rain showers from October through April (dry season). In our study areas, average monthly rainfall from May through September is over three times higher than from October through April for the period 1981–2010 (200.1 vs. 66.4 mm). Compared with these long-term averages, rainfall during our study was lower (162.0 vs. 55.8 mm), but broadly consistent between rainy (81.0% of long-term averages) and dry (84.0%) seasons, and thus likely to represent the normal effects of rainfall on mosquito seasonal patterns. Although year-to-year variation in rainfall may affect either dry-season egg mortality or rainy-season competition among mosquitoes, it is difficult to predict the outcomes of such variation due to the effects of rainfall on other environmental factors that may affect mosquito communities, including detritus resources.

There is little evidence that changes in temperature in the range experienced within and between seasons affect the outcome of competition among *A. albopictus, A. aegypti,* and *C. quinequefasiatus.* In the laboratory, [48] showed no effect of temperature in the 24–30°C range on the outcome of larval competition between *A. albopictus* and *A. aegypti*. A field study found reduced competition between *A. albopictus* and *A. aegypti* in the dry season in Florida, but this was almost certainly due to increased detritus resource inputs [21]. No studies that we are aware of have tested the effect of temperature on larval competition of *C. quinquefasciatus* with *Aedes* species [49]. A laboratory study showed greater reductions in survival and development rates from 20.0°C to 15°C for *A. aegypti* than *C. quinquefasciatus* [50]. However, while daily minimum temperatures can average below 15°C in southern Florida during the coldest months (i.e., January–March), brief periods (i.e., hours) at unfavorable temperatures are unlikely to affect the outcome of population-level competition between *C. quinquefasciatus* and *Aedes* compared to a lack of available habitat from lower rainfall. Moreover, temperatures from containers are likely to be higher than values from weather stations since containers are usually sheltered.

Although the observed greater proportional increase in *A. albopictus* from early to late rainy season is consistent with strong negative effects of interspecific competition on *A. aegypti*, we found that species co-occurrence did not reduce *A. aegypti* abundance per site. A prior field study showed *A. albopictus* and *A. aegypti* responded the same way to manipulations of egg desiccation and to seasonal differences in detritus inputs in the field, and that the effect of high detritus inputs in the dry season resulted in no detectable effects of competition in the dry season [21]. Similar responses to seasonal factors that reduce competition, such as more resources, suggest that season may contribute to coexistence as an equalizing mechanism [2] rather than a stabilizing mechanism. It is possible that *A. aegypti* can utilize specific container habitats that have relaxed interspecific competition at some sites.

Cemeteries were an important larval habitat for each species during the rainy season. While abundances of both *A. aegypti* and *A. albopictus* were lower in cemeteries during the early rainy season, cemeteries increased in importance by the late rainy season. Relative abundance of *C. quinquefasciatus* had the opposite relationship with cemeteries, which were important habitat early but not later in the rainy season. These results suggest that cemetery habitat plays a key role in mechanisms controlling the rate of increases in *Aedes* abundance and decreases in *C. quinquefasciatus* abundance from early to late season. The similar responses of *A. aegypti* and *A. albopictus* to cemetery habitat between early vs. late rainy season may be due to a number of ecological processes. Cemeteries likely provide numerous existing containers for each species. Even within one cemetery, cemetery vases vary considerably in their biotic and abiotic conditions [15], [51], and it is likely that these *Aedes* species choose to oviposit in containers with particular conditions, largely independently of the other species [2], [52]. Interspecific aggregation caused both by random processes and cuing on the environment may reduce the competitive impact of *A. albopictus* on *A. aegypti,* and facilitate the local coexistence of these species in cemetery sites [52]. Coexistence of *A. aegypti* with *A. albopictus* within the same cemetery may also be facilitated if *A. aegypti* avoids oviposition in vases already inhabited by *A. albopictus*. Some mosquitoes alter their oviposition behavior in response to conspecifics or to controphic non-mosquito larvae (e.g., [53], [54]) and conspecific eggs [55] already present in the habitat. However, we are unaware of any evidence for oviposition deterrence by other competing mosquito species, and this is an area for future research. Interspecific aggregation between *A. aegypti* and *A. albopictus* within cemeteries may be especially important for coexistence in the late rainy season when abundances of *A. aegypti* are highest.

During the late rainy season, cemetery was the only positive predictor of *A. aegypti* abundance at the 200-m scale; all other land cover variables were negative predictors of *A. aegypti*. The proportion of sites where *A. aegypti* was sampled (Table 2) declined 50% between the early and late rainy season, but this was predominantly due to loss of site-level occupancy in the urban areas, whereas the proportion occupancy in cemetery sites was relatively unchanged. However, the proportion of the replicate ovitraps where *A. aegypti* were present (Table 2, in parentheses) declined across all sites between early and late rainy season. [20] compared *A. albopictus* and *A. aegypti* abundances from oviposition traps placed in the same cemeteries vs. traps in the intervening urban matrix. [20] proposed that *A. aegypti* would be superior to *A. albopictus* at colonizing vacant cemeteries. In a metapopulation setting, this superior colonization ability would result in escape from regional exclusion via competition from *A. albopictus* [22], [23], but [20] found no difference in abundances of *A. aegypti* and *A. albopictus* between cemeteries and areas in the intervening urban matrix. The present study suggests that cemeteries may be important habitat for both of these *Aedes* species, and may act as patches of ideal habitat that support these species as metapopulations. Out of the 19 total cemeteries sampled in the late rainy season, and ignoring the presence of *C. quinquefasciatus,* ten were colonized by either *A. albopictus* or *A. aegypti,* four had both species, and five were unoccupied by these *Aedes* (Table 2). Future research may be needed to further investigate the utilization of cemeteries by these *Aedes* species, and the role of metapopulation dynamics among cemeteries for maintaining species coexistence, especially in the late rainy season.

Opposite to *A. albopictus* and *A. aegypti*, *C. quinquefasciatus* were positively predicted by cemeteries in the early rainy season and negatively predicted by cemeteries in the late rainy season. These field results are consistent with the competitive exclusion of *C. quinquefasciatus* from cemeteries as *A. albopictus* increasingly utilized cemeteries during the rainy season. However, seasonal responses of *C. quinquefasciatus* to rainfall and interspecific competition may be more complex and difficult to understand than with *Aedes.* In addition to above ground containers, *C. quinquefasciatus* commonly utilize a wide variety of above- and below-ground storm water treatment devices, such as wetlands, retention basins, and catch basins [56]–[58]. The regional coexistence of *C. quinquefasciatus* with the competitively superior *A. albopictus* and *A. aegypti* may depend on utilization of these ground habitats by *C. quinquefasciatus*, with container habitats acting as secondary low quality habitats, where these *Aedes* species are less likely to oviposit. However, wetland cover was negatively associated with *C. quinquefasciatus* abundances in this study, suggesting that that these habitats may not be an important source of individuals collected in our traps. *Culex quinquefasciatus* abundance was associated with High Intensity Developed and Open Cover Developed area, both of which likely harbor below ground catch basins that may be a source for individuals collected in our traps.

Local habitat segregation in oviposition between *A. albopictus* and *A. aegypti* has been well documented along rural to urban gradients, with *A. albopictus* generally positively related to rural variables and *A. aegypti* related to urban variables (e.g., [40], [59]). Habitat segregation between *C. quinquefasciatus* and *Aedes* species has been mainly studied among individual containers [45]. To our knowledge only [20] has examined spatial patterns of competing mosquitoes at the within-city scale and related them across different land uses in an entirely urban environment. [20] did not find *A. albopictus* and *A. aegypti* densities to be specifically associated with cemeteries, but instead differed among broad land use categories, with *A. aegypti* more abundant in ovitraps in residential areas compared to industrial and commercial areas. Patchiness of urban landscapes can contribute to the coexistence of *A. albopictus* with the inferior competitors *A. aegypti* and *C. quinquefasciatus,* especially given that *A. aegypti* and *C. quinquefasciatus* start the rainy season with the numerical advantage. *Aedes aegypti* and *A. albopictus* abundances were similarly associated with cemetery habitats and not predicted by other landscape variables at the 50-m scale, suggesting that *Aedes* coexistence is unlikely to be due to spatial partitioning at the scale of land use types used in this study. Rather *A. aegypti* may be able to escape competitive exclusion by ovipositing in uninhabited vases within cemeteries. Investigating species’ utilization among individual resident vases or other container habitats within cemeteries or other land uses was beyond the scope of this study, although past studies have found evidence that interspecific aggregation is likely be important for *A. aegypti* coexistence with *A. albopicitus* in Florida [52].

The results of this study are generally consistent with those of [20], a prior study in the same locations, as well as studies in other urban areas that have shown spatial and temporal differences among *A. albopictus* and *A. aegypti* (e.g., [23], [25], [60]). However, this study builds on prior studies in two important ways. First, it shows that seasonal patterns of *C. quinquefasciatus* oviposition into small containers show a trend opposite to that of co-occurring *Aedes* species. Second, this study examined specific land cover variables within each city and quantified their relative importance for explaining species abundances, as well as how the effects of seasonality on mosquito ecology varies in conjunction with land use.
